# Use of accelerometers to assess and describe trailer motion and its impact on carcass bruising in market cows transported under North American conditions

**DOI:** 10.1093/tas/txab216

**Published:** 2021-11-20

**Authors:** Carollyne E J Kehler, Daniela M Meléndez, Kim Ominski, Gary Crow, Trever G Crowe, Karen S Schwartzkopf-Genswein

**Affiliations:** 1 Department of Animal Science, University of Manitoba, Winnipeg, Manitoba, R3T 2N2, Canada; 2 Agriculture and Agri-Food Canada, Lethbridge Research and Development Centre, Lethbridge, Alberta, T1J 4B1, Canada; 3 Department of Mechanical Engineering, University of Saskatchewan, Saskatoon, SK, S7N5A9, Canada

**Keywords:** acceleration, bruising, compartments, cull cows, transport

## Abstract

Increased trailer motion, coupled with large accelerations and decelerations, has been associated with decreased carcass quality and increased stress indicators in cattle, sheep, and hogs. However, motion of livestock trailers has not been measured in North-American cattle semi-trailers over long distances (> 1000 km). The objectives of this study were to develop a practical method of measuring transport trailer accelerations, to describe the range of accelerations cattle are exposed to under North American conditions, and to conduct a preliminary analysis of trailer accelerations for each compartment and its effect on carcass bruising. The root mean square (RMS) of acceleration was measured at a sampling rate of 200 Hz in 3 orthogonal axes; x (vertical), y (front-to-rear), and z (lateral; side-to-side) by clamping an accelerometer to the cross beam below each of the five compartments of 8 trailers transporting a total of 330 animals (674 ± 33.3 kg BW) from an assembly yard to a processing facility. Journeys took place on separate days and ranged in duration from 13 to 15.7 h. The number and severity of bruises per carcass were determined prior to trimming for *n* = 290 carcasses and the number of bruises per carcass ranged between 0.38 and 12.75, whereas the bruising score per carcass ranged between 0.38 and 14.88. Mean number of bruises and severity of bruises (bruising scores were assigned according to size using a three-point scale: 1) ≤ 6.5 cm, 2) 6.5 to 12 cm, and 3) ≥ 12 cm and bruising severity was determined by applying the weighted score to each bruise according to bruise area) per carcass was 4.52 ± 2.43 (*n*) and 5.31 ± 2.84, respectively. Accelerations in commercial transport vehicles were found to range between 0.33 and 1.90 m/s^2^, whereas the mean RMS of acceleration for all trailers (*n* = 31 accelerometers) was 1.01 ± 0.32 m/s^2^, 0.72 ± 0.31 m/s^2^, and 0.97 ± 0.30 m/s^2^ for the x, y, and z axes, respectively. Horizontal acceleration was greatest in the nose, back, and doghouse compartments (*P* = 0.05), whereas lateral acceleration was greatest in the nose and back compartments (*P* = 0.08). Although the nose, back, and doghouse compartments had the highest RMS values for the lateral and horizontal axes, there were no significant relationships between bruising and acceleration. Replication of this research is required to further understand the relationships between trailer motion, carcass bruising, and overall animal welfare in cattle transported long distances.

## INTRODUCTION

The safety and welfare of market cows during transport are of particular concern to producers, transporters, and processors due to the increased risk of death, injury, and severe carcass defects in comparison to feeder or finished cattle (Strappini et al., 2010; González et al., 2012). A common carcass defect in market cows is bruising, which is the accumulation of blood and serum in the affected tissue due to rupture of blood vessels caused by trauma (Hoffman et al., 1998). Studies on carcass bruising in cattle have indicated that cull cows are more likely to pass through a livestock market, which entails more handling and transportation time, therefore increasing the risk of bruising (Strapinni et al., 2010). Holstein cattle have been reported to have greater bruising than beef breeds (Lee et al., 2017), whereas female carcasses have been reported to have increased risk of bruising compared to male carcasses of similar weight, possibly due to higher reactivity to stressors, greater body size with prominent protrusions, and lower body conditions (Mendoca et al., 2018).

Bruising has been associated with poor driving (Tarrant, 1990), rough handling (Grandin, 1981), transportation time (Huertas et al., 2010), cattle source such as farm, dealer, or livestock market (Jarvis et al., 1995; Weeks et al., 2002, Strapinni et al., 2010), sex (Jarvis et al., 1995), protruding objects in handling facilities (Grandin, 1980), improper space allowance during transport (Eldridge and Winfield 1988; Tarrant, 1990), and faulty slaughter facility design and skill of handlers (Weeks et al., 2002). Bruising not only causes substantial economic loss to the cattle industry but also contributes to reduced cow welfare (Broom, 2003; Strappini et al., 2013; Grandin 2018).

Acceleration includes motion such as shocks and jolts that could be experienced as a consequence of aggressive braking or cornering as well as vibrations during transit that can be caused by road conditions, the standing orientation of the cattle, vehicle suspension, trailer flooring, and truck speed (Randall, 1992; Gebresenbet et al., 2011). Both magnitude (m/s^2^) and a frequency (Hz) of accelerations can be measured to assess the potential impact on livestock or humans. Trailer motion can be measured through the use of accelerometers that record amplitude of trailer vibrations as a function of time. Many commercially available accelerometers store data for a limited period of time are expensive (>$1000) and/or must be wired directly to a power source and data-storage device or computer due to limited battery life and the large volume of data that are produced. These factors make them impractical for data collection under commercial conditions due to increased labor and time required for installation and the inability to modify the trailer or run wires within a large number of trailers transporting cattle for long periods. Therefore, novel strategies to measure trailer motion are necessary to establish the relationship between bruising and motion, and if apparent, implement training techniques to mitigate bruising severity. Therefore, the aim of this study was: 1) to develop a practical method of measuring trailer acceleration; 2) to characterize the range of accelerations cattle are exposed to on typical North American cattle trailers during journeys greater than 13 h; and 3) to conduct a preliminary analysis of compartment acceleration and its effect on carcass bruising in market cows. The hypothesis of this study was that acceleration could be accurately measured using self-contained battery-powered accelerometers attached to commercial transport trailers and that larger accelerations would result in an increase in carcass bruising. Accelerations could be an influencing factor for the severity of bruising in cows because of its direct relationship with driving conditions, which is already acknowledged as a risk factor for cattle welfare (González et al., 2012) and other welfare parameters in pigs (Perremens et al., 1998; Morris et al., 2021).

## MATERIALS AND METHODS

Data collection within this study was approved by the Animal Care Committee of Lethbridge Research and Development Centre (ACC 1042) according to the guidelines established by the Canadian Council on Animal Care (CCAC, 2009).

### Accelerometer Calibration

All 32 tri-axial X16-1C accelerometers used in the study were calibrated in a laboratory by comparing their output with the output of a highly sensitive uniaxial piezoelectric accelerometer model 352C65 (PCB Piezotronics, NewYork, USA) with a sampling frequency of 400 Hz. All accelerometers were rigidly attached to a hollow structural section, which was then bolted to a shaker-table, as described by Doranga et al. (2014). The shaker-table performed a frequency sweep from 1 to 20 Hz in 255 s. A 1-20 Hz range was selected as it best represented the typical frequency range experienced by cattle on a livestock trailer (Gebresenbet et al., 2011). During testing, the orientation of the hollow structural section was changed twice so that the acceleration could be collected in all three axes (x, y, z). The X16-1C accelerometers were set at a sampling frequency of 200 Hz with a dead band setting of 0.718 m/s^2^ and a dead band timeout of 60 s to match the settings used during actual data collection from the trailer. The 352C65 accelerometer was set at a sampling frequency of 400 Hz and the data were directly uploaded to a computer using VIBpoint Framework by Data Solutions. The mean RMS was calculated for each 15 s of data collected during the test creating 17 values used for comparison in each axes. The log of the RMS values was used in the calibration to account for unequal variances across the range of accelerometer readings.

### Transport vehicles

**Figure 1.**
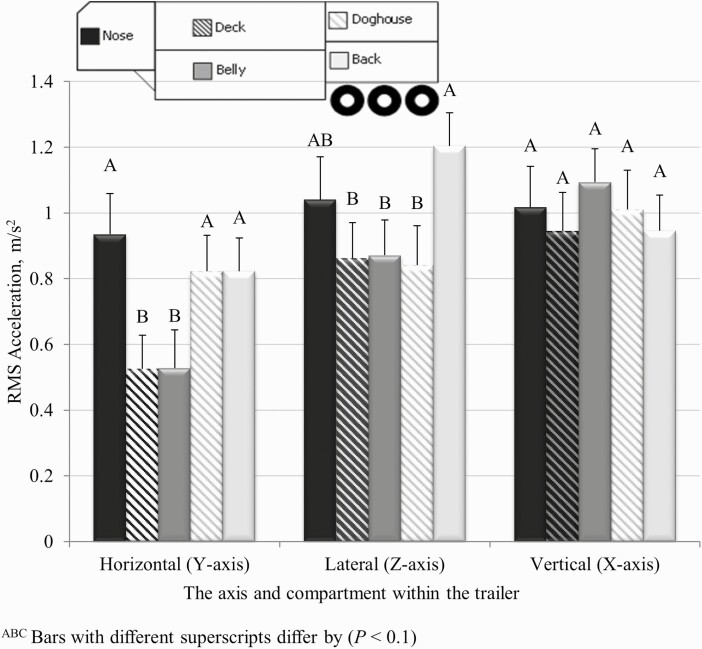
The least squares means (± SE) of the root mean square (RMS) in three axes measured in each of five compartments of a livestock semi-trailer transporting cull cows.

Eight commercial cattle transport trailers were used to record and assess trailer accelerations during transport. The trailers were constructed of aluminum with a drop center (pot belly) on the bottom level and air-ride suspension. Each trailer had five internal compartments: nose, deck, doghouse, belly, and back (Figure 1). Seven of the 8 trailers were of tri-axle configuration (Merritt Equipment Co., Henderson, CO; Wilson Trailer, Sioux City, IA; Dimensions: belly [17.2 to 19.5 m^2^], back [11.8 to 14.3 m^2^], deck [17.5 to 19.8 m^2^], doghouse [6.4 to 8.0 m^2^], and nose [7.7 to 8.2 m^2^]).

### Cattle and Transport Conditions

Three hundred twenty-nine market cows and one bull (674 ± 33.3 kg BW) were transported approximately 1,112 km in one of eight cattle trailers from southwestern Manitoba to the same slaughter facility in central Alberta. The number of cattle per trailer and loading densities ranged from 39 to 43 head and from 1.22 to 2.72 m^2^/animal per compartment, respectively. All cattle trailers followed the same route but were operated by different drivers. Radio frequency identification (RFID) tags of all animals were scanned at the time of loading to establish the relationship between location within the trailer and carcass bruising.

Each driver recorded the number of cattle in each compartment, departure and arrival time, condition of the cattle during the journey and reasons for stopping during transport. Journey duration ranged between 13 and 15.7 h, beginning between 06:11 and 07:17 mountain standard time (MST) and ending between 19:21 and 22:59 MST. The beginning of the journey started when the first animal entered the trailer and ended when the last animal was unloaded from the trailer.

### Accelerometers

Commercially available, tri-axial accelerometers (model X16-1C; Gulf Coast Data Concepts, Waveland, MS, USA) powered using a single AA lithium battery were used to measure g-force within each compartment of the trailers. The raw data were used to calculate the root mean square (RMS) of acceleration The accelerometers recorded acceleration in three orthogonal axes; x corresponded to the vertical axis, y the horizontal or front-to-rear axis, and z the lateral or side-to-side axis for the entire journey or until the battery life of the accelerometer was too low to continue recording. Due to environmental conditions (below freezing temperatures) and the need for a wireless accelerometer with an internal power source, the data could not be collected for the entire duration of the journey. Consequently, only data from the first half of each journey were used for data analysis.

The accelerometers were set at a sampling frequency of 200 Hz based on previous studies, indicating that it must be at least twice as large as the highest frequency movement being classified (Robert et al., 2009). The accelerometers had a dead band setting of 0.718 m/s^2^ and a dead band timeout of 60 s which were used to preserve battery life when acceleration levels were ≤ 0.718 m/s^2^. These parameters were selected after conducting a preliminary analysis. The incorporation of different levels of these settings were simulated using preliminary data, and settings were chosen to balance the need for sufficient detail to be retained in the recorded data while also allowing a reasonable amount of the entire trip to be recorded. Acceleration frequencies of interest on a cattle trailer are typically well below 100 Hz (Personal communication 2013, Trever Crowe, University of Saskatchewan) and therefore the 200 Hz sampling frequency was considered suitable. A frequency weighting of 1 was used on the acceleration data as the frequency weighting in cattle is unknown.

### Accelerometer Attachment

A 10-cm C-clamp was used to attach the X16-1C accelerometers to the trailer. A bolt was passed through the body of the accelerometer and the arm of the clamp, rigidly attaching the accelerometer to the clamp. Each accelerometer was fastened to a cross-beam underneath the middle (front-to-rear direction) of each of the five compartments of the trailer. The C-clamps were tightly attached to the beams, 10 cm from the right side-wall (passenger side in America) of the trailer. This method of attachment ensured a rigid connection between the trailer and accelerometer, facilitating installation and removal from the trailer

Using this technique, two accelerometers were located inside the trailer (on the ceiling above the belly and back compartments to assess the acceleration of the deck and the doghouse) and three accelerometers were attached outside the trailer (under the nose, belly, and back compartments). The accelerometers were wrapped in 15-cm long foam tubing to protect them from road debris (stones, mud, etc.) and tampering by the cattle.

### Testing Rigidity of Accelerometer Attachment

At the end of the journey, the attachment of each accelerometer was tested. If the C-clamps were moveable by hand but were still clamped to the beam, the data from these accelerometers were tested by performing a Fast Fourier Transform (FFT) on 20 s of acceleration data from three suspected loose and three securely attached clamps using Matlab R14 SP2 (MathWorks, Natick, MA). The resulting frequency spectrums were then interpreted and compared. Data from three loose accelerometers were then removed from analysis based on the visual recognition of peaks of acceleration at higher frequencies (80–90 Hz), which were inconsistent with the secure sensors.

### Carcass Data Collection

All cattle from each trailer (*n* = 290) were followed from unloading through processing and de-hiding until they reached the area within the plant located prior to carcass trimming. Assessment of bruising on each carcass was conducted by two trained observers on six locations (round, tail, right and left loin, and back anterior and posterior) according to the methods previously described by Goldhawk et al. (2015). Bruising scores were assigned according to size using a three-point scale: 1) ≤ 6.5 cm, 2) 6.5 to 12 cm, and 3) ≥ 12 cm (Hoffman et al., 1998). Bruising severity was determined by applying the weighted score to each of the bruises according to bruise area. All bruise scores (size and severity) recorded on the same carcass were summed to obtain a single score. The mean bruising score was determined for each compartment by dividing the value by the number of animals in the compartment. Only 7 out of the 8 trailers were assessed for bruising due to a delay in arrival of one truck to the plant. Animals were not assessed during the time that they were in lairage. Mounting and aggressive behaviors during this period of time could have caused bruising.

### Statistical Analysis

Statistical Analysis Software (SAS version 9.2, SAS Inst. Inc., Cary, NC,) was used to determine the descriptive statistics using the MEANS procedure and the regression equations for calibration using the REG procedure. The Mixed procedure in SAS 9.2 was used to determine the fixed effects of compartment (experimental unit) on both acceleration (*n* = 8) and bruising severity (*n* = 7) using a completely randomized block design with load as a random effect. Loading density was added as a covariate to the model but was removed because it was not significant (*P* value > 0.10). A quadratic multiple regression in SAS was used to determine the relationship between acceleration and bruising severity with load as a random effect. Linear regressions were performed to compare the output from the two types of accelerometers (X16-1C and 352C65) by determining the intercept and slope of the regression line. A general linear model (GLM) was used to determine if the slopes and intercepts varied between the two types of accelerometers. Kappa values for inter-observer reliability were 0.63 (*P* < 0.01) and the observers agreed on 99.5% of the bruise locations. Statistical significance was established at *P* < 0.05 and trends at 0.05 < *P* ≤ 0.11.

## RESULTS AND DISCUSSION

### Accelerometer and Methods

Calibration testing of the X16-1C accelerometers resulted in high coefficients of determination (> 0.99) for all (x, y, z) axes, indicating that they were comparable to the 352C65 calibration standard accelerometer. However, as significant differences between the slopes and intercepts were observed, unique regression equations for each accelerometer were developed using the REG procedure in SAS 9.2. and applied to all acceleration data prior to analysis. Our data suggest the X16-1C accelerometers appear to be an acceptable tool to assess trailer accelerations but require calibration with an accelerometer which has the capacity to accurately capture sampling frequencies of 400 Hz, like the 352C65, to allow for the development and use of unique regression equations which can be used to account for differences between device sensitivities and data offsets.

Due to limitations in the battery capacity of the sensors, acceleration was only measured for the first half of the journey for all but two journeys. The difference in RMS between the entire journey and the first half of those two journeys ranged between 7.22% and 14.54%. Enhanced battery life could remove the necessity of the dead band setting, making it simpler to apply frequency weighting (to account for changing sensitivities to vibration between animals) to acceleration data and include frequency in the analysis which would result in increased strength of analysis (especially when an accurate frequency weighting scale is determined for cattle). Use of a dead band setting could result in over-estimation of the RMS of acceleration because data falling below the threshold of the setting are not recorded. Although this could occur at any acceleration rate, most of the missing data points were around 0 m/s^2^. Therefore, although this effect is minimal, final RMS values may be impacted, suggesting that trailer accelerations would be more accurate if a dead band setting was not used. Advances in sensor technology that could extend battery life would be valuable for future long-distance transport research.

The mean RMS values were 1.01 ± 0.32 m/s^2^, 0.72 ± 0.31 m/s^2^, and 0.97 ± 0.30 m/s^2^, in the vertical, horizontal, and lateral axes, respectively. These values are similar to accelerations measured from the frame of a European livestock trailer traveling on three road types at four different speeds with mean RMS values of 1.52 ± 0.45 m/s^2^, 1.32 ± 0.37 m/s^2^, and 0.81 ± 0.12 m/s^2^ in the vertical, horizontal, and lateral axes, respectively (Gebresenbet et al. 2011). Cann et al. (2004) reported lower accelerations between 0.12 and 0.52 m/s^2^ RMS during highway travel; however, accelerometers were placed on the driver’s seat rather than on the frame of the trailer which could have dampened accelerations. Lateral accelerations between 0.69 and 2.74 m/s^2^ were recorded from a loaded North American tractor-trailer driving through a slalom maneuver on gravel roads at speeds between 18.5 and 34.8 km/h (Clark et al., 1999). Higher accelerations may be attributed to travel on gravel roads while continually turning, unlike the majority of travel in our study which occurred on straight, paved highways where traffic densities were low with little need for starting and stopping. Accelerations between 0.5 and 1.0 m/s^2^ have been described by humans as fairly uncomfortable, between 0.8 and 1.6 m/s^2^ as uncomfortable, between 1.25 and 2.5 m/s^2^ as very uncomfortable and greater than 2.0 m/s^2^ as extremely uncomfortable (Randall, 1992). For pigs, the welfare is considered compromised at 3 m/s^2^ (Perremans et al., 1998). Many of the RMS values in this study fell within the range that humans reported being very uncomfortable (1.2 to 2.5 m/s^2^); however, there may be differences in how bipeds and quadrupeds experience accelerations.

Considerable research has been conducted to observe human response to vibrations of varying magnitudes and frequencies. Accelerations can cause muscle fatigue, reduced stability, discomfort, and motion sickness in humans (Cann et al., 2004). Although most research on humans has been conducted using frequency-weighted RMS values to account for changing sensitivity to vibration as established by The International Organization of Standardization (ISO) 2631-1 (1997) standards assessing human discomfort at a single vibration magnitude (Zhou et al., 2014) frequency sensitivity has been observed to change at different magnitudes of vibration, indicating that the commonly used weighting scale should be updated (Zhou et al., 2014). Sensitivity is not only affected by the combination of frequency and magnitude, but can also be affected by body size and mass, age, gender, and change of posture or “biodynamics” (Randall, 1992; Zhou et al., 2014). Frequency weighting according to current standards could therefore inaccurately depict both human and cattle vibration sensitivity. It is especially difficult to determine the frequencies at which livestock are most sensitive because of their inability to communicate level of discomfort. Vibrations in trailers transporting pigs have been above the exposure action value and the exposure limit value thresholds established by the ISO, suggesting that pig welfare could be compromised during transportation (Morris et al., 2021). Several studies have examined the change in physiological indicators at different frequencies or measured resonant frequencies of commodities in transport. Perremens et al. (1998) observed higher acceleration magnitude (> 3m/s^2^) and specific acceleration frequencies (8 and 18 Hz) caused greater heart rate in transported pigs, which is an indication of increased stress. Resonant frequencies (a specific frequency at which the vibration oscillations increase in amplitude) have been established at 1.3, 5.1, 12.6, and 23 Hz in European cattle being transported in trailers (Gebresenbet et al., 2011). A frequency weighting of 1 was applied in the current study due to the lack of published literature regarding cattle vibration sensitivity and the risk of unintentionally biasing the data with an inaccurate weighting scale.

The degree to which the trailer floor either attenuates or amplifies the acceleration experienced by cattle was not measured in this study due to practical constraints. However, Gebresenbet et al. (2011) reported a transmission of acceleration from frame to floor of 55%–73% and from floor to cattle of 100%–158%. These findings suggest that acceleration experienced by the cattle could differ from those measured from the trailer frame because of attenuation of acceleration from frame to floor and amplification of acceleration from floor to cattle, depending on the axes. This also suggests that the transmission of vibration from the frame to the floor to the animal is influenced by the structure and fabrication techniques of the trailer. It is therefore reasonable to expect that the transmission to animals will be different in the various compartments of the trailer.

In addition to RMS, accelerations may also be described such as: crest factor (peaks of acceleration), vibration dose (cumulative effect that the acceleration magnitude has over time), resonant frequencies, and the power spectral density (combination of the frequency and magnitude of vibration over time) as described by Gebresenbet et al. (2011). The use of these values was restricted in the current study because a dead band setting was used on the accelerometers. Consequently, it is recommended that this setting be avoided so calibration can be conducted on the raw acceleration values rather than the RMS of acceleration allowing for a more comprehensive evaluation of the acceleration variables on livestock trailers and their effect on the livestock.

In the present study, horizontal acceleration was greatest in the nose, back, and doghouse compartments (*P* = 0.05), whereas the lateral acceleration was greatest in the nose and back compartments (*P* = 0.08), depicted in Figure 1. Vertical acceleration had no significant relationship with compartment (*P* = 0.86). The highest accelerations in the lateral and horizontal axes were in the nose, back, and doghouse compartments. Both the back and nose compartments are positioned directly over the tires of the trailer or tractor, the proximity to the tires can result in greater acceleration levels, whereas vibration toward the middle of the trailer is likely to be dampened. The nose compartment is also in direct contact with oncoming wind during transport and the tractor portion of the vehicle, potentially resulting in the observed elevated vibrations in these compartments. Contrary to our study, where no differences were observed for vibrations between compartments, vibrations were reported to be higher in the bottom deck compartments closer to the tires in trailers transporting pigs (Morris et al., 2021).

Horizontal acceleration for the doghouse and back compartments were not different. This is reasonable as the doghouse is directly above the back compartment and therefore both compartments are subjected to similar horizontal accelerations. Interestingly, lateral acceleration was greater in the back than in the doghouse compartment. The observed differences could potentially be attributed to movement of animals in the doghouse, as the accelerometer which was located on the roof of the back compartment was in close proximity to the floor of the doghouse. The doghouse could also be less affected by the tires that are immediately below the back compartment due to the weight and inertia of the load. However, this was not the case, as similar differences in lateral acceleration were not observed for the belly and the deck which were comparable in terms of accelerometer location between the two compartments. Therefore, similar readings in the belly and deck were likely less because of the flexibility and damping of the structure. Another explanation for the difference in lateral acceleration may be due to loading density as the average space allowance of the deck and the doghouse for all 8 trailers was 1.4 and 1.7 m^2^/animal, respectively. Therefore, it is possible that greater space allowance in the doghouse resulted in more animal movement, and increased lateral acceleration of the back compartment.

### Acceleration and Bruising

**Figure 2.**
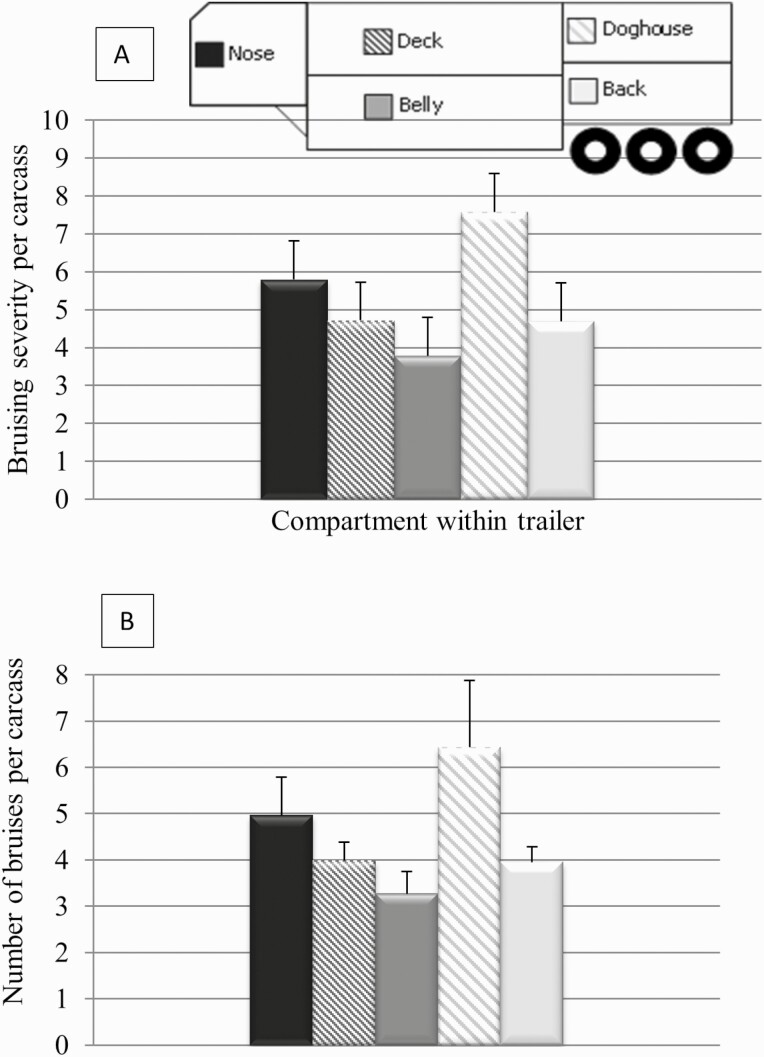
The least squares means (± SE) of (A) the bruising severity per animal and (B) the number of bruises per animal in each of five compartments of a livestock semi-trailer transporting cull cows (*P* = 0.11).

The doghouse tended to have higher bruising severity (7.58 ±1.01) and number of bruises than the other compartments (*P* ≤ 0.11), as described in Figure 2A and B. It should be noted that higher bruising in cattle loaded into the doghouse may have been caused by the fact that cattle must negotiate ramps and navigate a 180° turn to reach the doghouse, which is generally an L shape compared to the other compartments which are rectangles thereby increasing the risk of bruising. Although none of the axes of acceleration had a significant relationship with bruising; the greatest association was found in the lateral axis (*r*^2^ = 0.49; *P* = 0.25) followed by the horizontal (*r*^2^ = 0.17; *P* = 0.69) and vertical axes (*r*^2^ = 0.01; *P* > 0.97). To the authors’ knowledge, no published studies have examined the relationship between transport trailer motion (accelerations) and carcass bruising in cattle. It is important to note that other transport factors can be used to corroborate the impact of accelerations on bruising, especially in the lateral and horizontal axes, and their impact on livestock welfare. For example, vertical accelerations are more dependent on the speed of the trailer than the other axes. Air suspension systems, found on the majority of semi-trailers, could attenuate vertical accelerations and associated muscle fatigue and motion sickness caused by road conditions (Peeters et al., 2008; Gebresenbet et al., 2011). Magnitude of lateral and horizontal accelerations is impacted by driving style and cattle standing orientation (Peeters et al., 2008; Gebresenbet et al., 2011). Driving style is defined by the overall speed, speed of turns, and abruptness of starting and stopping (Peeters et al., 2008). Both Tarrant (1990) and Strappini et al. (2012) reported that the greatest incidences of loss of balance in cattle occurred during cornering and braking and that 3.8% of total bruising found on Chilean cows at slaughter was caused by cattle falling as a result of rough braking during transit. Furthermore, lateral accelerations have been identified as an important stressor for pigs, as measured by heart rate variability (Peeters et al., 2008). To maintain balance and reduce the impact of lateral and horizontal acceleration, standing orientation of cattle has been observed as perpendicular to the forward motion of the trailer (Tarrant, 1990; Gebresenbet et al., 2011). Maintaining balance is essential to avoid contact with other animals or the sides of the trailer thereby reducing the incidence of bruising (Strappini et al., 2012). Inappropriate use of sticks and prods during loading and unloading are risk factors which can cause bruising (Jarvis et al., 1995;Strapinni et al., 2013). Handling at the time of loading and unloading was not controlled in this study; therefore, bruising could have been due to handling during loading and unloading.

This is the first study, with acknowledged limitations, to examine the relationship between accelerations and bruising under North American commercial transport conditions. Additional research should focus on assessing transmission measurements between frame, floor, and cattle as well as exploring fixed levels of acceleration (high, medium, and low) under commercial conditions, while controlling stocking density, speed of the trailer, road conditions, number and speed of turns to more fully understand the impact of acceleration on bruising in road transported cattle. In the present study, data collection was limited to the first half of the journey due to the restricted battery life of the sensors. Lack of an association between bruising severity and accelerations could be due to the lack of information of the second half of the journey where accelerations could differ from the first part of the journey. Development of sensors with extended battery capacity is required to accurately assess the association of acceleration and carcass bruising.

## CONCLUSIONS

An important contribution of this study was the development of a methodology for utilizing commercially available accelerometers to measure motion in a livestock transport semi-trailer over long distances. These methods can now be applied to future commercial cattle research. Accelerations in commercial transport vehicles were found to range between 0.33 and 1.90 m/s^2^, and the mean vertical, horizontal, and lateral axes acceleration was 1.01 ± 0.32 m/s^2^, 0.72 ± 0.31 m/s^2^, and 0.97 ± 0.30 m/s^2^, respectively. The nose, back, and doghouse compartments had the highest RMS values in both the lateral and horizontal axes. In contrast, the vertical acceleration did not differ by compartment. Although no significant relationship between acceleration and bruising was observed, the association between these two parameters warrants further investigation. Advances in sensor technology that could extend battery life would be valuable for future long-distance transport research.
